# Race and Physical Education

**Published:** 1902-06-28

**Authors:** 


### The Hospital

A Journal of

The Medical Sciences and Hospital Administration.

Vol. XXXII.—No. 822. Edited by Sir Henry Burdett, K.C.B., and Solomon C. Smith, M.D., M.R.C.P. [June 28, 1902.

### Race and Physical Education.

The contrast between the Indian soldiers who are encamped at Hampton Court and the 'Arrys and 'Arriets who have "crowded on to stare," too often, we fear, in a manner to justify the rest of the quotation, "as men of courtesy untaught," is one which cannot be regarded with feelings of satisfaction. It is perfectly true that the magnificent orientals are the picked men, not only of their race, but also of their regiments, while the miserable little London cads who have gone to look at them would, in most instances, be the results of such a selection of the unfittest as only the most vacuous idleness could effect. It is perfectly true that a detachment of the guards, or a body of metropolitan policemen, would offer just as great a contrast to a chance collection of cotton operatives from the "chawls" of Bombay; but the one contrast is before us and is in evidence, while the other has to be conjured up by the imagination, and is not, at least in our time, likely to be realised. A contrast of a somewhat similar description, moreover, is afforded by many of the colonial troops who have come to us for the Coronation, and whose lithe figures and fine proportions are becoming familiar in our streets. It will be remembered that, at the commencement of the war now happily concluded, more than one very venomous letter was published by the *Times* paper from a correspondent who declared himself to be a Boer, and who founded his predictions of the speedy and complete defeat of our forces upon his impressions of the physical degeneracy of our people. These predictions have happily been falsified by the event; but, nevertheless, it would not be the part of wisdom to shut our eyes to any element of truth which may have been underlying them. The fact that we have been able to furnish a sufficiency of men of adequate physique does not at all justify us in regarding the physique of the nation as a matter which may be trusted to take care of itself; and we cannot afford wholly to neglect even the impression as to this matter which our visitors will be likely to take away with them. Few things are more calculated than stature and bearing to impress the imagination with a sense of superiority and of power, or to reconcile the many nationalities under our government to the sway which our people have come to exercise over them. Few things are more calculated to render that sway irksome, and even humiliating, than the presence in our streets of crowds of people, any one of whom the average Indian or Colonial soldier could break across his knee, or with whom, in the graphic language of Western America, it would be easy for him to "wipe up the floor."

Underlying the constant presence amongst us of the objectionable couple, once described by our contemporary the *Globe* as "the small young woman and the small young man," and underlying also the natural consequences of their friendships, we have a very grave and serious evil in the shape of the total neglect of physical education by the great majority of our people, and the abysmal ignorance of its value, its importance, and even of its nature, by which the average school manager or schoolmaster is characterised. In schools of the better class only too much attention is given to "games," but the attention is given to them as ends and not as means to that systematic physical training which, even in the most expensive schools, is too often utterly neglected. We are reaping the harvest of our past; and the average muddle-headed citizen thinks of education only as comprising the three R's, together with due precautions against the pupil being encouraged or permitted to think for himself on the most important subjects about which human thought can be occupied. The leading idea is to impart knowledge, not to train mental capacity; and it is scarcely recognised that even knowledge can be more easily imparted to a boy who fills his chest and squares his shoulders, than to one who sits huddled into a bunch. The stunted child, who never fills his chest and whose brain is never supplied with well-aërated blood, is tolerably certain to grow up a dunce, whatever anybody may try to teach him; while the child whose physical powers are cultivated will at least learn from experience, even if he learns in no other manner. Physical training should be so employed as to cultivate alertness; and alertness of body, if not an essential condition of alertness of mind, is at all events in an eminent degree contributory to it. The failure of our school boards is writ large upon our adolescent working population, especially upon our adolescent males. It is writ in slow movements, in round shoulders, in gaping mouths, in clumsy gait—all of them evidences that we have failed to make the best of our children that circumstances would allow. The present generation has witnessed the almost complete abandonment of the games and contests most conducive to alertness, the games and contests which involved personal antagonism. The contests of the village green were largely instrumental in making England what she once was—the wrestling, the single-stick, the running, the leaping. Our young people have relinquished them in order to loaf about while "professionals" bring football or cricket to a pre-ordained conclusion. It is indeed high time that we should "wake up," and that we should make our educators and our children wake up along with us.

